# Identification of Genetic Factors Controlling the Formation of Multiple Flowers Per Node in Pepper (*Capsicum* spp.)

**DOI:** 10.3389/fpls.2022.884338

**Published:** 2022-05-09

**Authors:** Youngin Kim, Geon Woo Kim, Koeun Han, Hea-Young Lee, Jinkwan Jo, Jin-Kyung Kwon, Zachary Lemmon, Zachary Lippman, Byoung-Cheorl Kang

**Affiliations:** ^1^Department of Agriculture, Forestry and Bioresources, Research Institute of Agriculture and Life Sciences, Plant Genomics and Breeding Institute, College of Agriculture and Life Sciences, Seoul National University, Seoul, South Korea; ^2^Vegetable Research Division, National Institute of Horticultural and Herbal Science, Rural Development Administration, Jeonju, South Korea; ^3^Inari Agriculture, Cambridge, MA, United States; ^4^Howard Hughes Medical Institute, Cold Spring Harbor Laboratory, New York, NY, United States

**Keywords:** pepper, flower production, yield, quantitative trait locus, genome-wide association study, genotyping-by-sequencing

## Abstract

Flower production provides the foundation for crop yield and increased profits. *Capsicum annuum* is a pepper species with a sympodial shoot structure with solitary flowers. By contrast, *C. chinense* produces multiple flowers per node. *C. annuum* accounts for 80% of pepper production worldwide. The identification of *C. chinense* genes that control multiple flowers and their transfer into *C. annuum* may open the way to increasing fruit yield. In this study, we dissected the genetic factors were dissected controlling the multiple-flower-per-node trait in *Capsicum*. 85 recombinant inbred lines (RILs) between the contrasting *C. annuum* ‘TF68’ and *C. chinense* ‘Habanero’ accessions were phenotyped and genotyped. Quantitative Trait Loci (QTL) analysis identified four novel QTLs on chromosomes 1, 2, 7, and 11 that accounted for 65% of the total phenotypic variation. Genome-wide association study was also performed on a panel of 276 genotyped and phenotyped *C. annuum* accessions, which revealed 28 regions significantly associated with the multiple-flower trait, of which three overlapped the identified QTLs. Five candidate genes involved in the development of the shoot and flower meristems were identified and these genes could cause multiple flowers per node in pepper. These results contribute to our understanding of multiple flower formation in *Capsicum* and will be useful to develop high-yielding cultivars.

## Introduction

Pepper (*Capsicum* spp.) is an agriculturally and economically important vegetable crop worldwide. The United Nations Food and Agriculture Organization (FAO) estimated that fresh and dried pepper production reached 36 million tons across the top 20 pepper-producing countries in 2020 (Countries by commodity, FAO^1^). Pepper is prized in various cuisines due to heat-causing capsaicinoids and nutritional benefits including a high content of pro-vitamin A (carotene), E (a-tocopherol), and vitamin C (ascorbic acid) (Penella and Calatayud, 2018). Pepper is also grown for its pain-killing and medicinal properties, for use in chemical industries, and as an ornamental plant (Fraenkel et al., 2004).

Yield, stress resistance, and fruit quality are three main traits driving crop breeding. Of these traits, improving crop yield is perhaps the most critical objective. Since the total number of flowers produced by a plant will determine the number of harvested fruits, the number of flowers per node (or per inflorescence) is a key target for higher yields. An increase in the number of flowers does not always increase the yield. As the number of flowers increases, so do the nutrients required for reproductive growth and fruit elongation, as well as nutrients for extra vegetative growth. Therefore, the more flowers there are, the more sensitive it is to the environment. Therefore, more sophisticated environmental control is required for high yield. Although the number of flowers does not necessarily result in high yields, it does create the possibility of high yields. The genetic factors determining flower number per inflorescence have been studied in cereals and horticultural crops such as rice (*Oryza sativa*) and tomato (*Solanum lycopersicum*) (Bai et al., 2012; Sasaki et al., 2017; Soyk et al., 2017a).

*C. annuum* is the most widely cultivated in the world among five domesticated *Capsicum* species: *C. annuum*, *C. chinense*, *C. frutescens*, *C. baccatum*, and *C. pubescens*. *C. annuum* exhibits a sympodial shoot structure with solitary flowers, whereas *C. chinense* produces multiple flowers per node. Therefore, one possible approach to increase pepper yield would be to identify *C. chinense* genes controlling the formation of multiple flowers and introduce them into *C. annuum*. Tanksley and Iglesias-Olivas (1984) established that the multiple-flowers-per-node trait in *Capsicum* is quantitatively inherited. However, the genetic mechanisms underlying this phenotype in pepper are poorly understood.

The identification of genetic factors governing quantitative trait loci (QTL) is commonly performed with bi-parental mapping populations, such as segregating F_2_s, backcross populations, and recombinant inbred lines (RILs) (Szalma et al., 2007). With recent advances in high-throughput sequencing and genotyping, a high-density genetic linkage map can be generated for the precise detection and characterization of QTLs. However, even with high-density genetic maps, conventional QTL mapping approaches are limited by (1) low mapping resolution and (2) the fact that they represent the variation between only two parental alleles, despite the breadth of allelic variation available from natural populations.

The limitations of traditional QTL analysis can be ameliorated by genome-wide association studies (GWAS), which test associations between nucleotide polymorphisms and phenotypic variations across large natural populations (Yano et al., 2016). GWAS may however generate false-positive associations between a phenotype of interest and unlinked markers due to a strong population structure. Although many statistically advanced models have been developed, spurious association peaks arising from population structure in crops cannot be easily controlled. Thus, combining GWAS and QTL analyses can at least partially compensate for the limitations intrinsic to each approach, allowing the identification of genes controlling agronomically useful QTLs with high confidence.

In this study, QTL analysis was performed for the trait of multiple flowers per node in one interspecific *Capsicum* RIL population derived from parental accessions with contrasting phenotypes. To ensure accurate linkage analysis, a high-density genetic map was generated. In a complementary approach, 276 *C. annuum*-clade accessions, including *C. annuum*, *C. chinense*, and *C. frutescens*, genotyped-by-sequencing (GBS) and phenotyped for the same trait to conduct GWAS. By comparing the physical locations of the QTLs from traditional QTL mapping and GWAS, three common QTLs were identified. Notably, the five candidate genes are known to control inflorescence meristem development. These results demonstrate that the complementary gene search of QTL mapping and GWAS using two populations is efficient and that higher yields might be possible if these genes are introduced into commercial peppers to breed new varieties.

## Materials and Methods

### Plant Materials

An interspecific population of 85 recombinant inbred lines (RILs) at the F10 to F12 generation was derived from a cross between the *C. annuum* accession ‘TF68’ (female recipient) and the *C. chinense* accession ‘Habanero’ (pollen donor) by single-seed descent (Kang et al., 2001; Kim et al., 2010). This population is referred to as TH RIL according to the names of its founding parents. The ‘TF68’ accession sets a single flower at each node, whereas the male parent, ‘Habanero,’ normally bears two to four flowers per node (Supplementary Figure 1). All TH RILs were cultivated in three biological replicates in the FarmHannong greenhouse in Anseong, South Korea, 2017.

Cold Spring Harbor Laboratory (CSHL) *Capsicum* core collection was used for GWAS. To reduce population structure, the accessions *C. baccatum* and *C. pubescens* were excluded. 276 accessions (98 *C. annuum* accessions, 66 *C. chinense* accessions, 67 *C. frutescens* accessions, and 45 *Capsicum* spp. accessions) were phenotyped for the presence of multiple flowers per node, growing at least four plants per accession in the upland farm, Riverhead field or in the greenhouse at CSHL in New York, the United States in 2015 (this latter experiment was conducted in the laboratory of Dr. Zachary Lippman; the collected phenotypes for the CSHL population were used under permission).

### Investigation of Flower Number and Imaging of the Shoot Apical Meristem

We collected data on the number of flowers per node after flowers were set or distinguished at the six nodes for the TH RILs, and then calculated the mean flower number by dividing the total number of flowers by the number of studied nodes and the number of replicates (Supplementary Table 1). For the CSHL population, the mean numbers of flowers were calculated from the first to the third node with a minimum of four biological replicates per accession.

**FIGURE 1:**
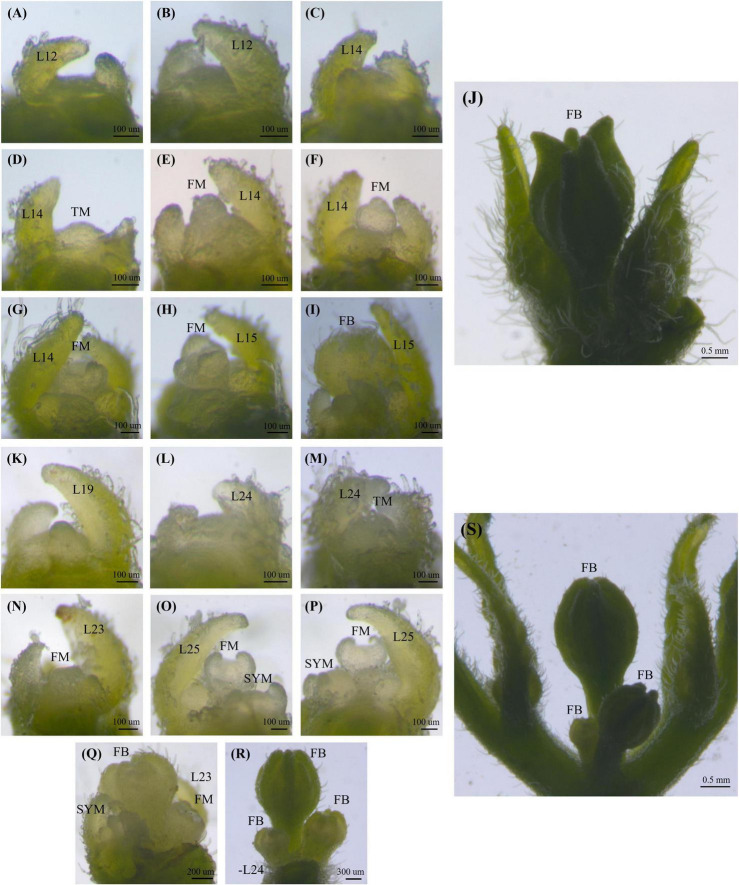
Shoot apical meristem and flowering pattern in the ‘TF68’ **(A–J)** and ‘Habanero’ **(K–S)** parental accessions. Leaf number is indicated by L, the transition meristem stage is marked by TM, the flower meristem is marked by FM, flower buds are marked by FB and the sympodial meristem is indicated by SYM.

To capture images of shoot apical meristems (SAM) on a stereomicroscope (Discovery.V12, Carl Zeiss, Germany), shoot apices of all genotypes were dissected from the seedling stage to the stage of flower bud development. Older leaf primordia (larger than 150 mm) were trimmed off under a stereomicroscope. SAM images were taken immediately after dissection with an integrated digital camera (AxioCam MRc, Carl Zeiss, Germany) attached to the stereomicroscope (Figure 1).

### Extraction of Genomic DNA and RNA

Genomic DNA (gDNA) was extracted from healthy young leaves from all TH RILs, the ‘TF68’ and ‘Habanero’ parental lines, and F_1_ plants using a modified cetyltrimethylammonium bromide (CTAB) method (Porebski et al., 1997). Leaf tissues were homogenized using 3 mm steel beads in a TissueLyser II sample disruptor (Qiagen, Netherlands). The quantity and purity of the purified gDNA were measured on a Nanodrop spectrophotometer (BioTek, Winooski, VT, United States) and by gel electrophoresis on 1.2% agarose gels. The confirmed DNA was diluted to a final concentration of 50 ng/μL with distilled water.

RNA was extracted from cotyledons. Dissected tissues were immediately frozen in liquid nitrogen and ground to a fine powder. Total RNA was extracted using the MG Total RNA Extraction kit (MGmed, South Korea) following the manufacturer’s instructions. RNA samples were eluted in RNase-free water (MGmed, South Korea). After isolation, the quantity and purity of RNA were determined on a Nanodrop spectrophotometer and checked RNA quality by gel electrophoresis on 1.2% agarose gels. First-strand complementary DNA (cDNAs) was synthesized from 2 μg of total RNA using the EasyScript Reverse Transcriptase kit (TransGen, China) with oligo(dT) primers. The resulting cDNAs were used to confirm cDNA sequence variation and for expression analysis.

### Construction of Genotyping-by-Sequencing Libraries and Data Analysis

Genotyped-by-sequencing libraries were constructed for all TH RILs by digesting gDNA with the restriction enzymes *Pst*I and *Mse*I using the SBG 100-Kit v2.0 (Keygene N.V., Wageningen, Netherlands). The gDNA was separately digested for the CSHL population with *EcoR*I and *Mse*I (Han et al., 2018). Then, adapters were ligated to the digested gDNA. The libraries were amplified by PCR with selective primers, which added the barcode GA for TH RILs and TA for the CSHL population. The amplified libraries generated from all TH RILs and two biological replicates for each parent were pooled in a single tube. The libraries for the CSHL population were pooled in three tubes each including at most 96 samples. All pools were sequenced on separate lanes on a HiSeq 2000 platform (Illumina, San Diego, CA, United States) at Macrogen (Seoul, South Korea).

All raw sequencing reads were trimmed to a minimum length of 80 bp and filtered for a minimum quality of Q20. Then, the filtered reads were aligned to the *C. annuum* ‘Dempsey’ reference genome v0.1 (unpublished) using the Burrows-Wheeler Aligner (BWA) program v0.7.12 (Li and Durbin, 2009). To call and filter the single nucleotide polymorphisms (SNPs), UnifiedGenotyper from GATK v.3.8-0 was used (DePristo et al., 2011). The SNPs from the TH RIL population were filtered for a minimum genotype quality of Q30 and minimum coverage of three reads.

The above described procedure was adjusted to call SNPs for the CSHL population, and with the same criteria. The mono or multiallelic SNPs and SNPs with a calling rate <0.6 were removed from the entire population. After filtering all SNPs, the missing data were imputed by using Beagle v4.1 and filled in the missing SNPs. Finally, an additional filtering step was performed based on minor allele frequency >0.03, and an inbreeding coefficient (F) > 0.8.

### Bin Map Construction for the Biparental TH Population

Missing data and non-polymorphic SNPs were eliminated from the genotypic data of the TH RIL population and the parental lines. Then, the recombination breakpoints were detected for all RILs using a modified sliding-window approach (Han et al., 2016). The ratios of SNPs were calculated between the two parental genotypes for each window, defined as 20 linked SNPs, and the overall ratio within each window was used to determine the genotype. SNP ratios >0.7 were scored as maternal of paternal, and SNP ratios between 0.3 and 0.7 were scored as heterozygous. The linkage map of the bins was constructed with the help of the Carthagene software (De Givry et al., 2005). The criteria used to construct linkage groups were a logarithm of the odds (LOD) threshold score of 3.0 and a limit distance between bins of 30 cM. The Kosambi genetic mapping function was used to calculate the distances between bin markers. The genetic bin map of TH RILs was constructed based on the *C. annuum* ‘Dempsey’ reference genome and compared to the physical distances from the reference genome in R.

### Quantitative Trait Loci Analysis of the Multiple-Flower-per-Node Trait

The phenotypic data collected from the TH RILs were used to detect quantitative trait loci (QTL) controlling the multiple-flower-per-node trait. Composite interval mapping was used using the collected phenotypic data and the high-density genetic bin map in Windows QTL Cartographer v2.5 (Wang et al., 2012). The genome-wide significance LOD threshold level was determined by running 1,000 permutation tests with a *p* < 0.05 probability level. The additive by additive effect between QTLs was measured using a multiple-interval mapping with a Bayesian information criterion (BIC-X) model. Then, the QTLs were mapped from the genetic to the physical map and a confidence interval was defined with a 99% probability.

### Genome-Wide Association Study for the Multiple-Flower-per-Node Trait, Population Structure, and Haplotype Block Estimation

For association mapping, 156,589 filtered SNPs detected from the 276 accessions of the CSHL population were used. PCA to estimate the population structure, calculation of kinship matrixes, and compressed mixed linear GWAS were conducted using the R package ‘Genomic Association and Prediction Integrated Tool (GAPIT)’ with default options (Lipka et al., 2012). Significant SNPs were determined as *P*-values calculated by the Bonferroni correction.

The ggfortify library in R was used to plot the CSHL population structure estimated by PCA. The haplotype blocks were estimated in the CSHL population using PLINK v1.9 (Chang et al., 2015; Tang et al., 2016) with the following settings: –blocks no-pheno-req no-small-max-span –blocks-inform-frac 0.9 –blocks-max-kb 2000 –blocks-min-maf 0.05 –blocks-recomb-highci 0.80 –blocks-strong-highci 0.85 –blocks-strong-lowci 0.7 –no-parents –no-sex.

### Candidate Gene Prediction and Sequence Variation Analysis

The candidate genes were selected which were closely linked to SNPs exceeding the significance threshold for association with the multiple-flower-per-node trait. Genes known to be involved in inflorescence development were also identified and selected as candidate genes if mapping within our QTL intervals. The annotated ‘CM334’ v1.6 reference genome (Annuum v2.0) was used for gene prediction rather than the ‘Dempsey’ reference genome v0.1 (Kim et al., 2014). To determine the physical locations of significant SNPs in the ‘CM334’ v1.6 reference genome, a Basic Local Alignment Sequence Tool (BLAST) search was performed using 450 kbp of genomic sequence centered on significant SNPs from the ‘Dempsey’ reference as a query against the ‘CM334’ v.1.6 reference. After obtaining the location of significant SNPs in the ‘CM334’ v1.6 reference genome, closely located and significant SNPs within a 2 Mbp interval at each locus were grouped and searched through annotated genes.

For candidate gene analysis, DNA sequences for each candidate gene were obtained from the NCBI database and blasted against the *C. annuum* ‘Dempsey’ v0.1 reference genome. Primers were designed based on the sequence from the ‘Dempsey’ accession. PCR was performed according to the following conditions: 35 cycles at 98°C for 10 s, 60°C for 30 s, and 68°C for 1 to 3 min depending on gene length, with PrimeSTAR GXL DNA polymerase (TaKaRa, Japan). PCR amplicons were separated on 1.2% agarose gels and purified using the LaboPass PCR clean-up kit (Cosmo Genetech, South Korea). Standard Sanger sequencing was conducted at Macrogen (Seoul, South Korea) and analyzed with the Lasergene SeqMan program (DNASTAR, Madison, WI, United States). Then, candidate gene sequences were compared across multiple accessions to measure the extent of sequence variation: *C. annuum* ‘Dempsey’ v0.1 and ‘CM334’ v1.55 (accessions with a single flower per node, like the parental line ‘TF68’) and *C. chinense* ‘PI159236’ v1.2 (accession with multiple flowers per node, similar to the ‘Habanero’ parental line) (Kim et al., 2017).

### Expression Analysis of Candidate Gene

To determine the expression level of the candidate gene *SP5G* in the control of the multiple-flower-per-node trait, real-time quantitative polymerase chain reaction (RT-qPCR) was conducted using a LightCycler^®^ 480 Instrument II (Roche Molecular Systems, Inc., Pleasanton, CA, United States). The cDNAs, synthesized from RNA samples extracted from the cotyledons of the parental lines ‘TF68’ and ‘Habanero’ in biological triplicates, were used as templates for RT-qPCR. RT-qPCR was performed according to the conditions: 45 cycles of 95°C for 10 s, 58°C for 20 s (ramp rate 2.2°/s), and 72°C for 20 s by using Ex Taq DNA polymerase (TaKaRa, Japan), followed by melting curve analysis with the conditions 95° for 5 s, 65° for 1 min (ramp rate 2.2°/s), and detection (97°, ramp rate 0.11° /s, and acquisition 5 s per °) (Figure 7). The relative expression was determined with the ΔΔC_*t*_ method between ‘TF68’ and ‘Habanero,’ using *Actin* as reference gene.

### Sequence-Characterized Amplified Region Marker Development for Candidate Gene

Sequence-characterized amplified region (SCAR) markers were designed for *SP5G* based on variation in gene structure. Primers were also designed for genotyping with Primer3web (v.4.1.0^2^) (Supplementary Table 2). SCAR markers were used to genotype the CSHL population to validate the results obtained from the trait association. For SCAR marker analyses, PCR was conducted according to the conditions: 32 cycles of 98°C for 15 s, 57°C for 15 s, and 72°C for 3 min. Resulting amplicons were separated on a 1.2% agarose gel.

## Results

### Variation for Number of Flower per Bud and Shoot Apical Meristem Development

The ‘TF68’ accession produced on average 1.1 flowers per node, as determined from the first to the sixth inflorescence nodes, whereas the ‘Habanero’ accession carried three times as many flowers, with 3.3 flowers per node. In the 85 TH RILs used for QTL mapping, the average flower number per node was 1.49. The flowers-per-node phenotype of the CSHL population was also determined from the first to the third nodes and it ranged from 1.0 to 3.4 with an average number of 1.32. Each *Capsicum* species exhibited distinct flower numbers per node, with an average of 1.02 flowers per node in *C. annuum* species, 1.33 in *C. frutescens* species, and 1.87 in *C. chinense* species, which corresponds to single, intermediate, and multiple flowers per node, respectively.

The shoot apical meristem (SAM) first gives rise to leaves before transitioning to making flowers. The TF68 accession showed a mean leaf number until the reproductive transition and flowering of 12.0 in the spring, and 13.9 in winter. The ‘Habanero’ accession took longer to reach the reproductive stage, with a leaf number ranging from 18 to 25 leaves. During our characterization of the meristems, transition meristems (TMs) were broader than vegetative meristems; the last leaf formed by TMs was also smaller than a typical leaf (Figures 1D,M). As plants matured, TMs turned into floral meristems (FM), taking on the classic apical dome shape before differentiating directly into a flower (Figures 1E,N). The ‘TF68’ accession normally produces a single flower per node, and we determined that the TM in ‘TF68’ plants developed a single FM finally forming a single terminal flower (Figures 1E–J). By contrast, the TM in the ‘Habanero’ accession gradually developed multiple FMs in one node as seen during a time-series analysis of meristem development (Figures 1N–S).

### Bin Map of the Biparental Population

**FIGURE 2:**
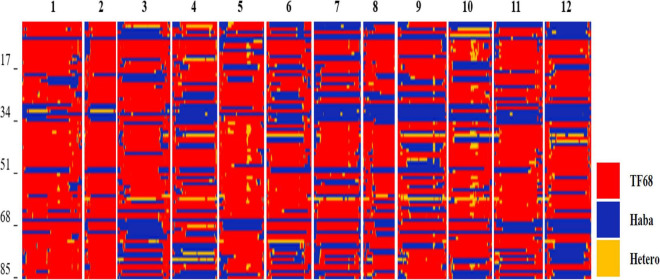
Bin map of the TH RIL population. Red indicates regions with a TF68-type genotype, blue represents Habanero-type genomic regions, and yellow denotes heterozygous regions.

All TH RILs were genotyped-by-sequenced (GBS). GBS libraries were prepared from *Pst*I/*Mse*I-digested genomic DNA. An average number of reads per sample were about 4 million and 10,851 SNPs were detected by aligning all reads to the *C. annuum* ‘Dempsey’ reference genome (Table 1). SNPs tended to be more densely concentrated at the ends of the chromosomes than around the centromeric regions (Supplementary Figure 2A). Using a modified sliding window approach (Han et al., 2016), a high-density bin map of the TH RIL population was constructed (Figure 2). The map consisted of 1,789 bins with a mean inter-bin distance of 0.96 cM (Table 2). Each of the 12 linkage groups matched one of the 12 *Capsicum* chromosomes, with chromosome 12 having the longest genetic distance and chromosome 8 the shortest. The total length of the linkage map was estimated to be 1,713 cM. The linkage map was compared to the *C. annuum* ‘Dempsey’ reference genome to determine the physical position of each bin (Supplementary Figure 3). Overall, the genetic and physical positions of the bins appeared collinear.

**TABLE 1:** Number of sequencing reads generated from GBS and SNPs from GWAS and QTL mapping.

Summary statistic	TH-RIL	CSHL population
# of accessions (lines)	85	276
Genotyping method	GBS (*Pst*I/*MseI*)	GBS (*EcoR*I/*Mse*I)
Avg. # of reads per sample	4,103,757	1,936,524
Total # of SNPs	10,851	156,589
Avg. inter-SNP interval (bp)	278,746	19,351

*The symbol “#” indicates the number.*

**TABLE 2:** Summary of bins on each chromosome of the TH RIL population by GBS.

Chr.	Number of SNPs	Number of bins	Physical length of bins (Mbp)		Genetic distance of bins (cM)	
			Mean	Total	Mean	Total
1	1,253	192	1.73	332.8	0.78	149.6
2	925	148	1.19	175.8	0.85	126.3
3	1,321	203	1.43	291.2	0.85	171.7
4	854	150	1.66	248.4	0.78	116.4
5	801	143	1.75	250.2	1.04	148.8
6	930	145	1.72	249.5	0.87	125.7
7	896	130	2.02	262.9	0.92	119.7
8	744	113	1.53	173.4	1.00	112.7
9	839	142	1.91	271.6	0.80	113.3
10	711	141	1.70	240.1	1.21	170.8
11	723	138	1.96	271.2	1.04	143.8
12	854	144	1.79	257.7	1.49	214.1
Total	10,851	1,789	1.69	3,024.7	0.96	1,712.9

### Quantitative Trait Loci Mapping for the Flower Number per Node

**FIGURE 3:**
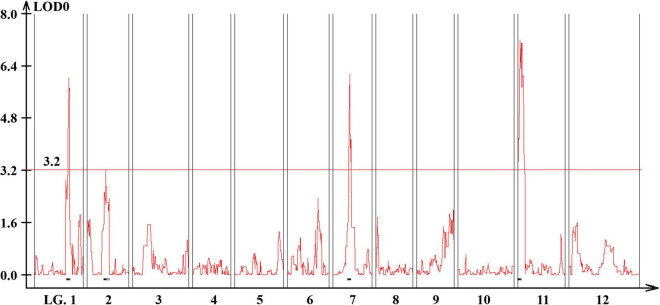
Genome-wide QTL plot associated with the multiple-flowers-per-node trait detected in the TH RIL population. The red horizontal line indicates a LOD score threshold of 3.2. Small black bars above the *X*-axis indicate the detected QTLs.

Quantitative trait loci landscape was explored which were controlling the flower number per node in TH RILs (Figure 3). The high-density bin map of all 85 RILs was combined with the phenotypic data to identify QTLs: significant QTLs were detected on chromosomes 1, 2, 7, and 11, which were referred to as *TH-mfx*, where x is their chromosome number (Table 3). *TH-mf11* showed the highest LOD score and explained 8.13% of the total phenotypic variation. The physical locations of each detected QTL in the TH RILs were determined using the *C. annuum* ‘Dempsey’ reference genome. *TH-mf1* was located between 172.9 and 191.4 Mbp on chromosome 1, *TH-mf2* mapped to 128.6–139.6 Mbp on chromosome 2, *TH-mf7* was between 16.0 and 32.1 Mbp on chromosome 7, and *TH-mf11* mapped to 1.6–3.2 Mbp on chromosome 11.

**TABLE 3:** Quantitative trait locis controlling the multiple-flowers-per-node trait detected across TH RILs.

Trait	QTL	Chr.	Location (cM)	LOD	R^2^ (%)	Direction*	Additive effect
Multiple flower	*TH-mf1*	1	102.1–106.9	6.05	6.10	–	0.2
	*TH-mf2*	2	53.7–65.1	3.23	3.03	–	0.11
	*TH-mf7*	7	48.3–52.3	6.15	6.45	–	0.17
	*TH-mf11*	11	3.3–8.4	7.19	8.13	–	0.18

*Genotypes that increase the multiple flower per node phenotype. – means the genotype resembles that of ‘Habanero’ (multiple flowers per node).

**FIGURE 4:**
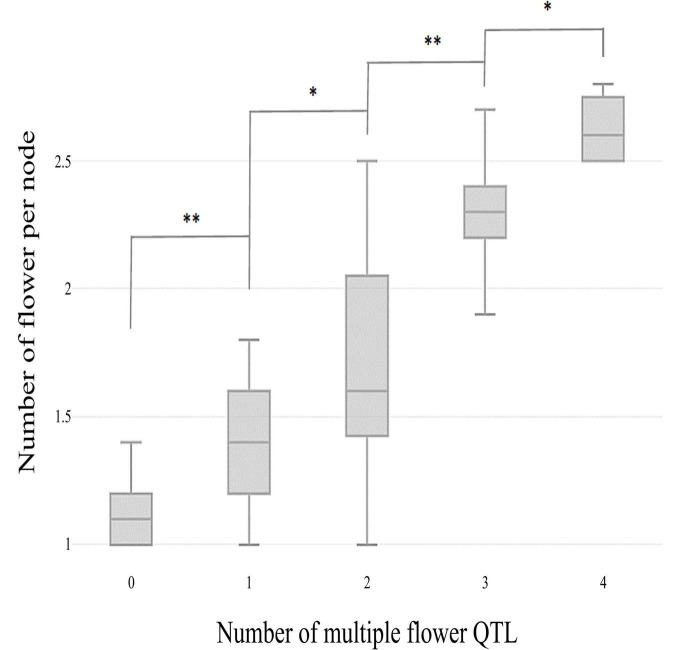
Box plot distributions of the multiple flowers per node phenotype regulated by four *TH-mf* QTLs in plants from the TH RIL population. The numbers on the *x*-axis indicate the number of candidate QTLs homozygous for the ‘Habanero’ allele. Asterisks indicate significant differences (*P < 0.05 and ^**^P < 0.01). *P*-values were determined by a two-tailed, two-sample *t*-test.

The skewed distribution of the flower number per node in TH RILs indicated that there may be epistatic interactions between the QTLs (Supplementary Table 1). Thus, epistatic effects were measured between the four detected QTLs by performing multiple-interval mapping (MIM). However, any meaningful additive-by-additive epistasis between QTLs was not detected. Then, the effects associated with each QTL were calculated by MIM (Table 4). The four QTLs identified here explained 65.0% of the observed phenotypic variation. To validate the effect on the multiple flowers phenotype of the detected QTL, the TH RILs were sorted based on their genotypes at the markers most closely linked to each QTL, and plotted the flower number measured in the resulting subset of accessions as a box-plot. The four QTLs identified here were significantly associated with differences in the number of flowers per node in the TH RILs (Figure 4).

**TABLE 4:** Cumulative effect of all QTLs detected for the multiple-flower-per-node trait in TH RILs.

Trait	QTL	R^2^ (%)*
Multiple flower	*TH-mf1*	26.9
	*TH-mf2*	3.6
	*TH-mf7*	18.5
	*TH-mf11*	16.0
	Total	65.0

*R^2^ value of individual QTL was evaluated by MIM analysis.

### Single Nucleotide Polymorphism Filtering and Haplotype Block Analysis of the Cold Spring Harbor Laboratory Population for Genome-Wide Association Studies

Genome-wide association studies were performed for the multiple-flowers-per-node trait using 276 *C. annuum*-clade accessions, comprising 98 *C. annuum*, 66 *C. chinense*, 67 *C. frutescens*, and 45 unidentified *Capsicum* species. As for QTL mapping, all accessions were genotyped-by-sequencing. Each sample produced around 2 million sequencing reads. SNPs were identified by aligning the reads to the *C. annuum* ‘Dempsey’ reference genome. After filtering SNPs for minor allele frequency, inbreeding coefficient, and calling rate, 156,589 SNPs were used for further analysis. The SNPs in the GWAS population were evenly distributed, with a mean distance between SNPs of 19,351 bp (Supplementary Figure 2B and Supplementary Table 3).

**FIGURE 5:**
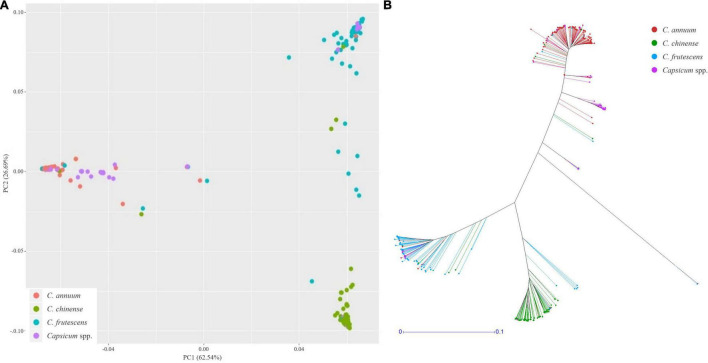
Population structure analysis of the CSHL population, with a principal component analysis **(A)** and a phylogenetic tree **(B)** determined from 156,589 SNPs. Red, green, sky blue and purple colors indicate *C. annuum*, *C. chinense*, *C. frutescens* and *Capsicum* spp. accessions, respectively.

Using these SNPs, a principal component analysis (PCA) divided the 276 *Capsicum* accessions into three subgroups (Figure 5A), which were supported by phylogenetic analysis (Figure 5B). These results showed that the accessions in the CSHL population were grouped according to their expected species groups: *C. annuum*, *C. chinense*, and *C. frutescens*. Fifteen accessions did not cluster with any of the subgroups. The population structure determined from the PCA was then applied for GWAS. Haplotype blocks were calculated along each chromosome using PLINK v1.9 with more relaxed settings than the default settings. About 60% of SNPs were grouped into 5,373 blocks, and each block contained 3–112 SNPs with a mean SNP number per block of 17.8 (Supplementary Table 3). Block sizes varied from 2 bp to 2 Mbp, with a mean block size of 450 kbp (range: 366 to 483 kbp), which is larger than the mean distance between SNPs of ∼19.4 kbp used for GWAS. Haplotype-based GWAS was then employed to reduce the false positives that can occur with SNP-based GWAS (Hayes, 2013).

### Genome-Wide Association Studies for the Multiple-Flowers-per-Node Trait

156,589 SNPs were used for GWAS predictions of the multiple-flowers-per-node trait using 276 *C. annuum*-clade accessions. A total of 83 SNPs exceeded the false discovery rate (FDR) threshold that was considered significantly associated with the trait of interest (Supplementary Figure 4). These SNPs were grouped into 41 genomic regions based on haplotype block estimation. To identify the corresponding physical locations of significant SNPs in the ‘CM334’ v1.6 reference genome, 450 kbp intervals were identified around each SNP from the ‘Dempsey’ reference genome and the matching sequences were searched in the ‘CM334’ v.1.6 reference genome. To avoid missing genes located near trait-associated SNPs and to cover all significant SNPs that were not included within a haplotype block, closely located significant SNPs within a 2 Mbp interval at each locus were grouped (Supplementary Table 4). Using gene annotation data (‘Annuum.v.2.0.chromosome.gff3’) and these grouped SNP loci, 388 genes, located in 28 associated regions, and their functions were identified.

**FIGURE 6:**
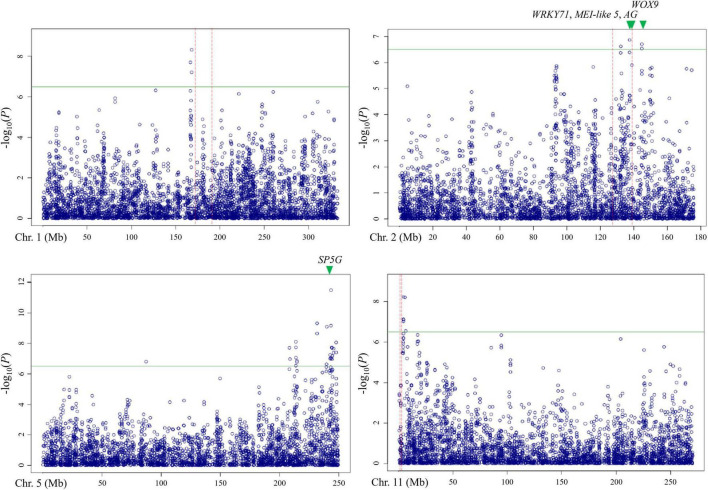
Comparison of QTL regions from the TH RIL population and Manhattan plots from GWAS using the multiple-flowers-per-node trait. The threshold of the –log(*P*) was 6.2. QTL region detected by QTL mapping are marked by red dashed lines, with green triangles indicating the position of the candidate genes.

Out of these 28 regions, three regions on chromosomes 1, 2, and 11 overlapped with the QTLs detected by bi-parental QTL mapping with the TH RIL population (Figure 6). On chromosome 1, one locus was physically located 4 Mbp away from *TH-mf1*, which is relatively close considering that the size of the *Capsicum* genome is over 3 Gbp. In addition, two significant SNPs were located within the *TH-mf2* QTL region. Likewise, a locus detected on chromosome 11 was only 800 kbp away from the *TH-mf11* region.

### Prediction and Expression Analysis of Candidate Genes Controlling the Multiple-Flowers-per-Node Trait

The combination of QTL and GWAS results allowed us to define a shortlist of high-confidence candidate genes involved in the formation of multiple flowers per node (Table 5). Out of the 28 associated genomic regions from GWAS, the most highly significant region on chromosome 5 corresponded to *SP5G* (*SELF PRUNING 5G*), named after the tomato homologue, also related to Arabidopsis *FLOWERING LOCUS T*, both known to control plant architecture and flower production. Another gene, *WUSCHEL-RELATED HOMEOBOX 9* (*WOX9*) (*CA.PGAv.1.6.scaffold79.74*), is a major determinant of inflorescence architecture in the tomato and was located about 600 kbp away from four significant SNPs located at 144.7 Mbp on chromosome 2 (based on the ‘Dempsey’ reference) (Lippman et al., 2008).

**TABLE 5:** Candidate genes for the multiple-flowers-per-node trait in the reference genome.

Gene annotation	Gene ID (CA.PGAv.1.6.)	Chr.	Physical location in CM334 v1.6 (bp)		Physical location in Dempsey (bp)	
			
			Start	End	Start	End
*WUSCHEL-related homeobox 9* (*WOX9*)	*scaffold79.74*	2	129,246,544	129,248,628	144,102,139	144,104,229
*MEI2-like 5*	*scaffold257.50*	2	134,623,870	134,633,408	137,762,619	137,771,713
*putative WRKY71*	*scaffold257.32*	2	134,965,138	134,966,803	137,481,716	137,483,298
*Floral homeotic protein AGAMOUS*	*scaffold411.1*	2	138,038,471	138,050,630	139,475,699	139,487,957
*FLOWERING LOCUS T* (*SELF PRUNNING LOCULS T; SP5G*)	*scaffold1310.12*	5	234,808,906	234,812,234	241,815,139	241,818,453

The comparison of QTL mapping and GWAS results led to the identification of 63 genes within co-located regions. Among them, the co-located region on chromosome 2 was associated with three genes encoding the floral homeotic gene *AGAMOUS* (*CA.PGAv.1.6.scaffold411.1*), *MEI2-like 5* (*CA.PGAv.1.6.scaffold257.50*), and a putative *WRKY71* homologue (*CA.PGAv.1.6.scaffold257.32*). These genes are known to function in shoot apical meristem development and/or floral meristem initiation and mapped to 139.4, 137.7, and 137.4 Mbp, respectively on chromosome 2 in the ‘Dempsey’ reference.

**FIGURE 7:**
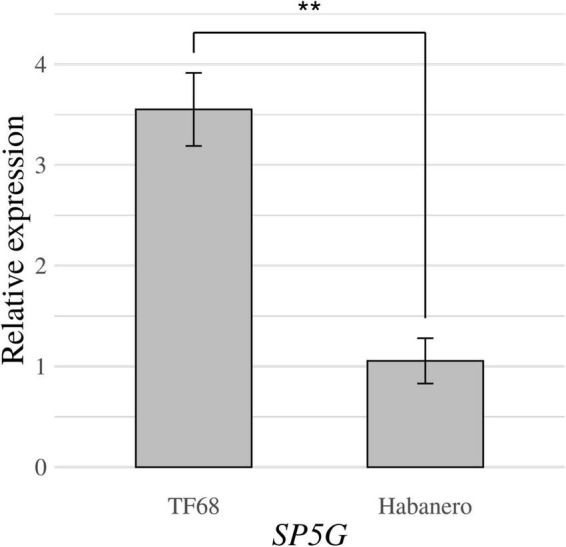
Relative expression of the *SP5G* gene in the parental accessions ‘TF68’ and ‘Habanero,’ as determined by RT-qPCR. Asterisks indicate significant differences (**P < 0.01). *P*-value was determined by a two-tailed, two-sample *t*-test.

RT-qPCR was conducted to compare the expression of the *SP5G* candidate gene between the ‘TF68’ and ‘Habanero’ accessions. The relative expression of SP5G was over three times higher in ‘TF68’ than in ‘Habanero,’ a difference that was highly significant (*P*-value = 0.005, Student’s *t*-test) (Figure 7). Therefore, the ‘TF68’ accession, which is early-flowering and bears a single flower per node, exhibits a much higher *SP5G* expression than the later flowering, multi-flower per node ‘Habanero’ accession.

### Confirmation of Quantitative Trait Loci and Genome-Wide Association Studies Analysis

To validate the role of the candidate genes listed in Table 5 in the multiple-flowers-per-node trait, Plants from the TH RILs were grouped according to their genotypes at the pepper homologues to *SP5G*, *AGAMOUS*, *WRKY71*, and *MEI2-like 5*. The bin markers, TH2-65.9 (located 100 kbp away from *AGAMOUS*), TH2-64.1 (8 kbp away from *WRKY71*), and TH2-62.9 (27 kbp away from *MEI2-like 5*) were used. From GWAS analysis, a sequence-characterized amplified region (SCAR) marker was designed for *SP5G* based on a 589 bp deletion in the first intron in the ‘Habanero’ accession that is also present in the *C. chinense* ‘PI159236’ v1.2 reference genome (Supplementary Figure 5A and Supplementary Table 2). Then, the CSHL and TH RIL populations were genotyped with the *SP5G* SCAR marker (Supplementary Figure 5B). Individual plants in the TH RIL and CSHL populations were classified into three groups according to their genotypes at *SP5G*: ‘TF68,’ heterozygotes, and ‘Habanero’ genotype, marked as A, H, and B, respectively (Figure 7).

**FIGURE 8:**
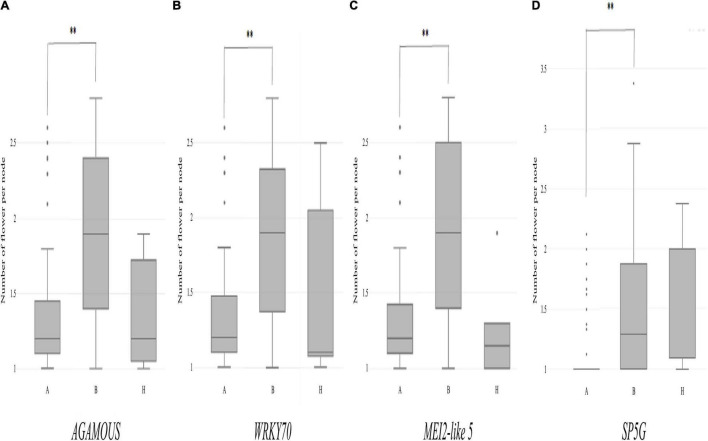
Distribution of the variation measured in the multiple-flowers-per-node trait associated with the genotypes of individual candidate genes in the TH RIL **(A–C)** and CSHL **(D)** populations. TH RILs were classified based on the genotype of the bin marker most closely linked to the target genes. CSHL *C. annuum* clade accessions were genotyped at the *SP5G* gene with the *SP5G* SCAR marker. Asterisks indicate significant differences ^**^P < 0.01. *P*-values were determined by a two-tailed, two-sample *t*-test.

Then, individual TH RILs were separated according to their genotypes at the three candidate genes: the genotype at these genes (or their closely linked SNPs) exhibited significant differences in their multiple flowers per node index, with the ‘Habanero’ allele always increasing flower number relative to the TH68 allele (Figures 8A–C). Similarly, in the CSHL population, differences in the genotype at *SP5G* led to highly significant differences in the number of flowers per node, with again the ‘Habanero’ allele resulting in more flowers per node when compared to the TH68 allele (Figure 8D).

### Sequence Variation of Candidate Genes

To explore the sequence variation in the candidate genes, their genomic sequences were extracted from the reference pepper genome. The extracted sequences were compared including the single flower per node accessions ‘Dempsey’ and ‘CM334,’ as well as the multiple flowers per node accession ‘PI159236.’ The changes of amino acids were detected in the predicted proteins encoded by *MEI2-like 5* and *WOX9*. For *MEI2-like 5*, six amino acid differences were discovered between ‘CM334’ and ‘PI156236,’ while *WOX9* contained three nonsynonymous substitutions in ‘Dempsey’ and ‘PI159236’ (Supplementary Data 1 and 2). Direct sequencing of *SP5G* from cDNAs synthesized from the ‘TF68’ and ‘Habanero’ parental lines revealed two SNPs in the coding region. However, both point mutations were synonymous mutations (Supplementary Data 3). The other floral homeotic gene *AGAMOUS* also had SNPs in the coding region between the ‘Dempsey’ and ‘PI159236’ reference genomes, but they did not result in amino acid changes. *WRKY71* showed no sequence variation in its coding region.

## Discussion

### Identifying the Multiple-Flowers Trait in Pepper

The number of flowers carried on each node varies depending on the *Capsicum* species. Identifying the genetic factors controlling this phenotype, and introgressing this trait into elite varieties may increase fruit yield potential. According to a previous report, the multiple-flowers-per-node trait is controlled by several loci (Tanksley and Iglesias-Olivas, 1984). Precise mapping of quantitative traits has been challenging due to the difficulties associated with phenotyping over multiple years and environments and the need for high-density genetic maps. However, the advent of high throughput next-generation sequencing (NGS)-based genotyping technologies, together with the development of statistically advanced QTL prediction models, provide unprecedented opportunities to identify genes controlling desirable quantitative traits. In this study, we attempted to identify the genetic factors associated with the production of multiple flowers per node in *Capsicum* using QTL mapping and GWAS.

### Detecting Regions of Multiple-Flower Genes Using Quantitative Trait Loci Mapping and Genome-Wide Association Studies

A comparison of the physical locations of the main QTL peaks obtained from traditional QTL mapping with our GWAS results highlighted three overlapping regions on chromosomes 1, 2, and 11. In a separate study, Zhu et al. (2019) mapped QTLs associated with the multiple-flowers-per-node trait on chromosomes 2, 7, and 10 using a high-density genetic map from a population of 150 F_2_ plants derived from a cross between *C. chinense* ‘740’ and *C. annuum* ‘CA1.’ Out of their three QTLs, the QTL on chromosome 2 showed the largest effect and explained about 40% of the standing variation. To estimate the physical location of the chromosome 2 QTL identified by Zhu et al. (2019) on our own map, the ‘Dempsey’ sequence corresponding to the candidate gene was used underlying this QTL as a query in a BLAST search. The underlying candidate gene was located in the 159–160 Mbp region on chromosome 2, which is 20 Mbp away from *TH-mf2* and 14 Mbp away from significant SNPs identified by GWAS. Zhu et al. also observed two novel minor QTLs on chromosomes 8 and 11 with other interval mapping methods; due to a lack of information for QTLs other than the one on chromosome 2, we were not able to compare our data with theirs.

### Prediction of Candidate Genes Controlling the Multiple-Flower Trait

By combining GWAS and QTL analyses, five candidate genes were identified as regulators of the multiple-flowers-per-node trait in pepper: *WOX9, MEI2-like 5, WRKY71, AGAMOUS*, and *SP5G*. All candidate genes are known to have functions in SAM development, and as such determine plant and inflorescence architecture. Looking at homologues of our candidate genes in other members of the Solanaceae family like a tomato can help validate functional assignments. *WOX9* shows similarity to the meristem maintenance gene *WUSCHEL* (*WUS*); both are plant-specific transcription factors. Lippman et al. (2008) determined that *WOX9* (also named *COMPOUND INFLORESCENCE* and *S*) is a major determinant of inflorescence architecture in tomatoes. Mutant alleles of *S* dramatically increase branch and flower numbers (Lippman et al., 2008; Soyk et al., 2017a). The *S* homologue in pepper was described by Cohen et al. (2014) and named *Capsicum annuum S* (*CaS*). The *CaS* mutant shows delayed initiation of sympodial growth or termination of sympodial meristems and completely inhibits flower formation (Cohen et al., 2014). Since *WOX9* in pepper promotes meristem transition from vegetative to reproductive phase and is required for flower formation, it likely contributes to the control of multiple flowers per node. Also, three amino acid changes were detected between ‘Dempsey’ and ‘PI159236’ at *WOX9*.

The function of *MEI2-like 5* has been studied in maize in some detail (*Zea mays*). When *Terminal ear 1* (*Te1*) (the maize *MEI2-like* homologue) is mutated, *te1* plants have a smaller vegetative shoot apex and fewer leaf founder cells (Veit et al., 1998). The authors hypothesized that the small size of the SAM in *te1* mutant stemmed from more frequent leaf initiation relative to the wild type (Anderson et al., 2004). Furthermore, the normally long vegetative internodes on the main shoot that precede the tassel are abnormally short, with the tassel being either less branched or feminized (Anderson et al., 2004). Genetic variation in *MEI2-like 5* may also modulate SAM activity in pepper.

More recently, *WRKY71* was shown to accelerate the initiation of the floral meristem by activating *FLOWERING LOCUS T* (*FT*) and *LEAFY* expression in Arabidopsis (Yu et al., 2016). Tomato *SP5G*, the *FT* homologue in this species, represses flowering (Cao et al., 2016). *SP5G* controls the flowering time of both primary and canonical axillary shoots and is the major contributor to photoperiod sensitivity in wild tomatoes (Lemmon et al., 2018). Therefore, mutations in *SP5G* cause rapid flowering and enhance the compact determinate growth habit of tomato plants (Soyk et al., 2017b). However, in the orphan Solanaceae crop groundcherry (*Physalis pruinosa*), a mutation in the *SP5G* orthologue (*Ppr-SP5G*) results in more fruits on each branch, but not in early flowering on the primary shoot (Lemmon et al., 2018). Indeed, gene-edited *Ppr-sp5gCR* mutant plants have 50% more fruits per shoot than non-edited controls. Groundcherry is more distantly related to pepper than tomato (Wolff, 1991; Särkinen et al., 2013). However, the plant architecture and sympodial growth habit of pepper are much closer to those of groundcherry than of tomato. As revealed by previous work, phenotypic differences can occur when orthologous *FT* family members are mutated due to species-specific sympodial growth patterns. Therefore, we suggest that *WRKY71* and *SP5G* are strong candidates that influence the multiple-flowers-per-node trait. Another candidate gene on chromosome 2 is an *AGAMOUS* homologue, which encodes a MADS-box domain transcription factor essential for the termination of floral stem cell fate. Mutations in this gene terminate floral stem cell maintenance in Arabidopsis by indirectly repressing *WUS* (Liu et al., 2011).

## Conclusion

In conclusion, combining the conventional QTL mapping and complementary GWAS, we demonstrated that the five candidate genes *WOX9*, *MEI2-like 5*, *WRKY71*, *AGAMOUS*, and *SP5G* are strong candidates for controlling the formation of multiple flowers per node in pepper. Although functional investigations are required to confirm the functions of these genes, a deep understanding of the genetic architecture of the multiple-flowers-per-node trait may help in the breeding of pepper varieties for higher yield.

## Data Availability Statement

The original contributions presented in the study are publicly available. These data can be found here: National Center for Biotechnology Information (NCBI), accession numbers ON081481–ON081485.

## Author Contributions

YK cultivated TH-RILs and performed phenotyping, genotyping of the TH-RILs and the CSHL core collection, QTL analysis, and GWAS. GK performed TH-RIL phenotyping, expression analysis, and manuscript writing. KH constructed the TH-RILs. H-YL constructed the core collection of pepper. JJ helped the genotyping. J-KK supervised the GWAS. ZLe and ZLi constructed the CSHL core collection and made phenotypic data. B-CK supervised the overall processes and revised the manuscript. All authors contributed to the article and approved the submitted version.

## Conflict of Interest

The authors declare that the research was conducted in the absence of any commercial or financial relationships that could be construed as a potential conflict of interest.

## Publisher’s Note

All claims expressed in this article are solely those of the authors and do not necessarily represent those of their affiliated organizations, or those of the publisher, the editors and the reviewers. Any product that may be evaluated in this article, or claim that may be made by its manufacturer, is not guaranteed or endorsed by the publisher.

## Footnotes

1
https://www.fao.org/faostat/en/#rankings/countries_by_commodity

2
http://bioinfo.ut.ee/primer3/
