# Supplementary figures and images for: Identification of Genetic Factors Controlling the Formation of Multiple Flowers Per Node in Pepper (Capsicum spp.)
Source: Front Plant Sci. 2022 May 9;13:884338. doi: 10.3389/fpls.2022.884338 (PMC9125326; doi:10.3389/fpls.2022.884338)

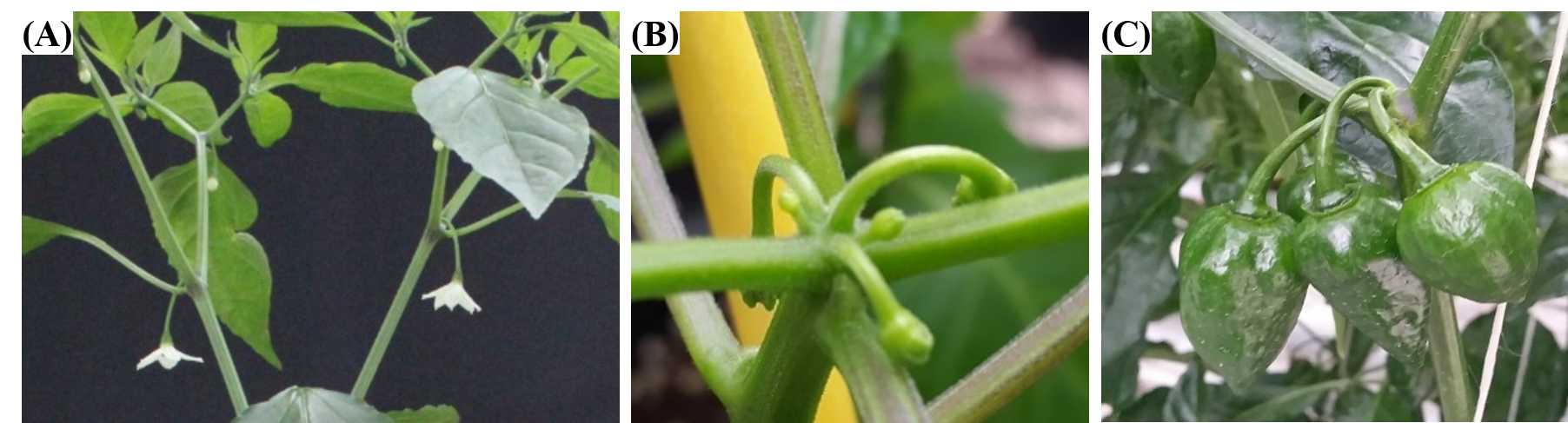

Supplement: Supplementary file 1 [file Image_1.TIF]

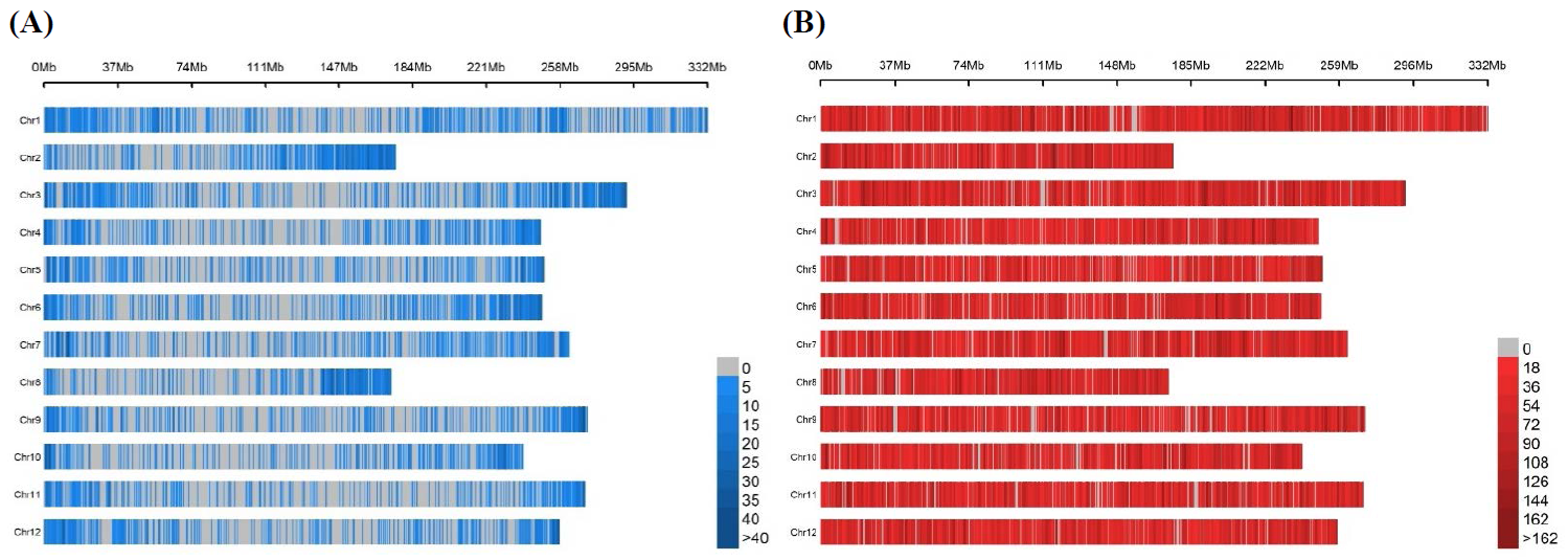

Supplement: Supplementary file 2 [file Image_2.TIF]

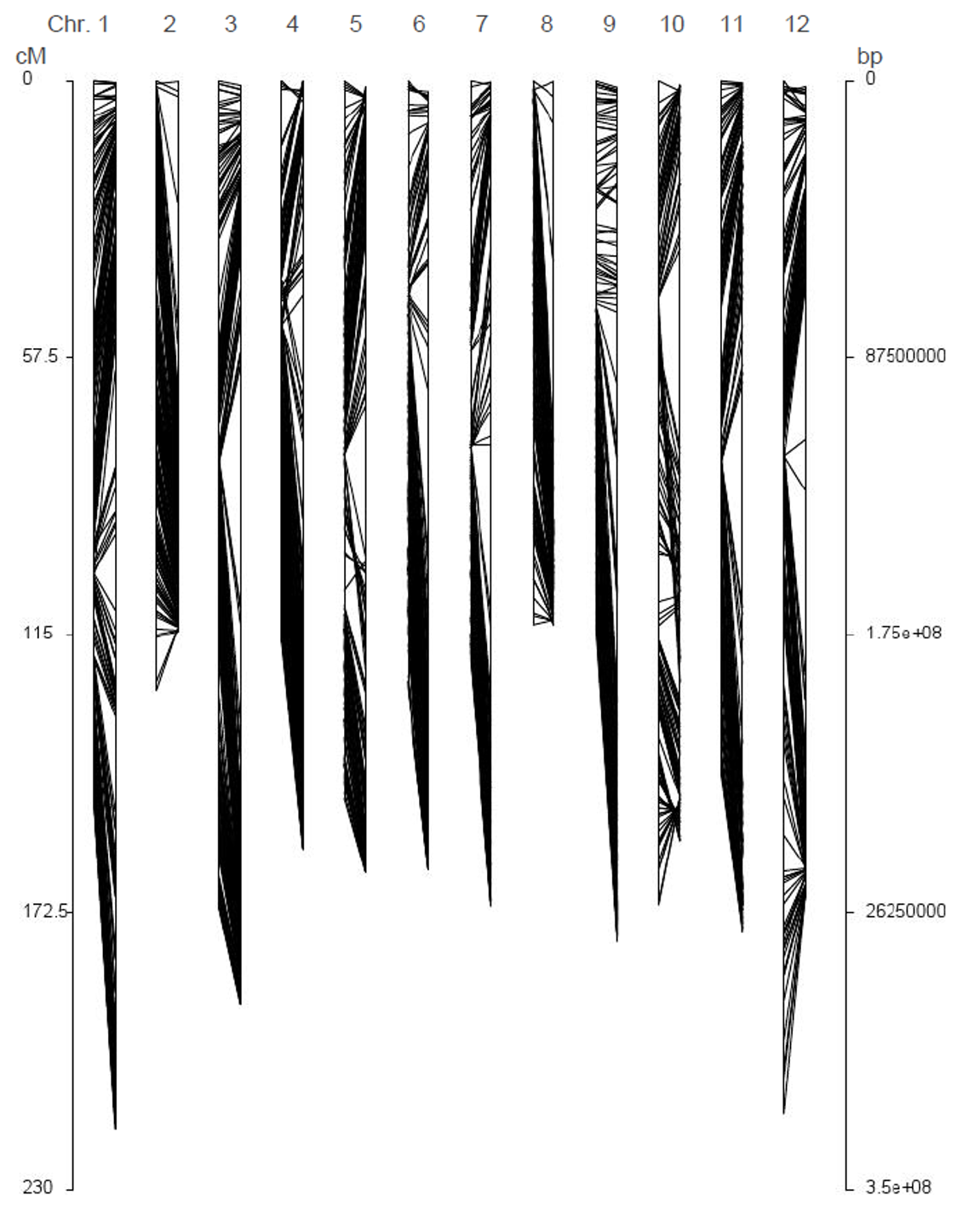

Supplement: Supplementary file 3 [file Image_3.TIF]

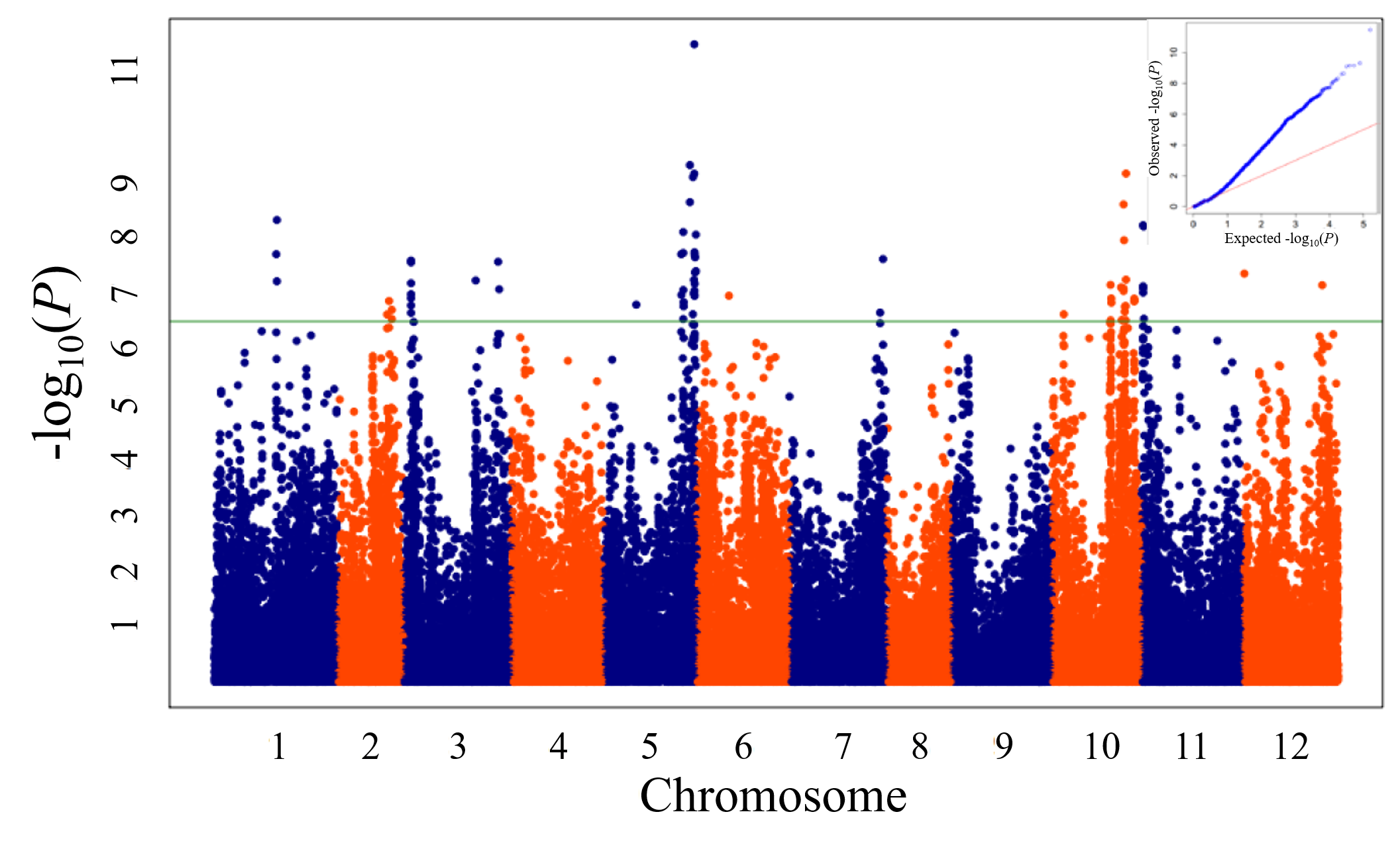

Supplement: Supplementary file 4 [file Image_4.TIF]

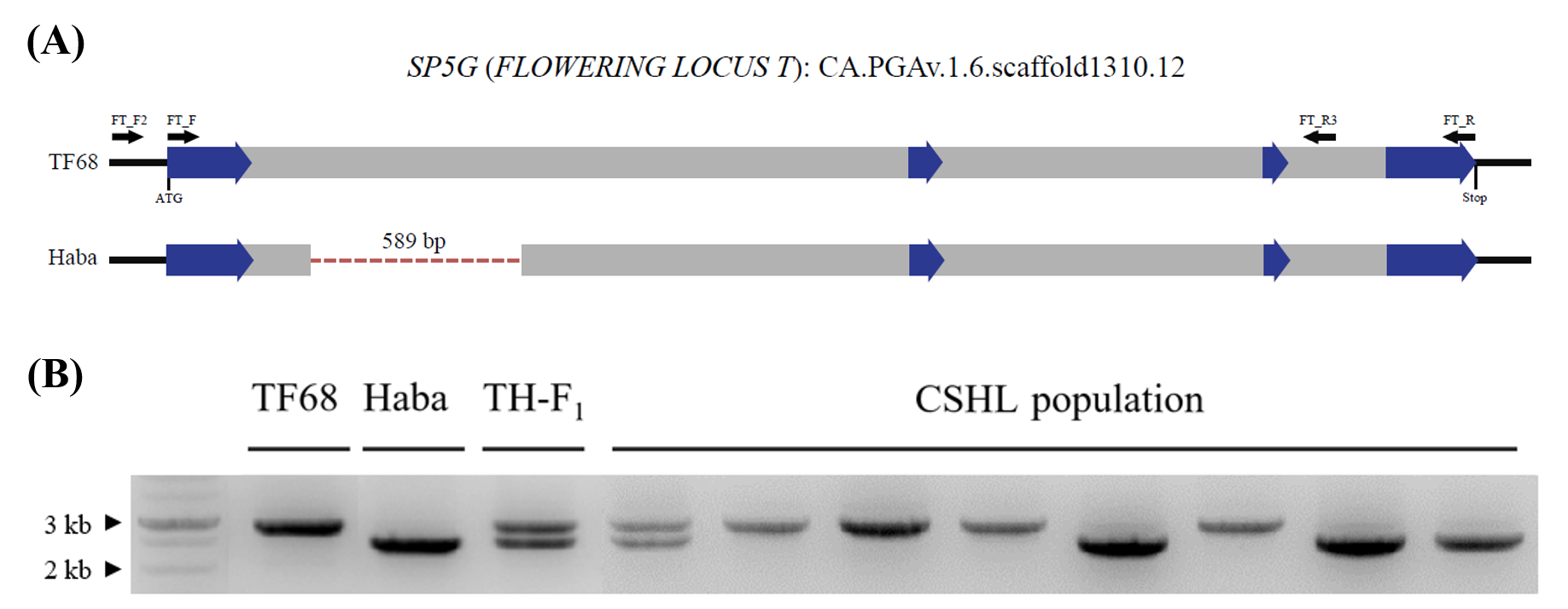

Supplement: Supplementary file 5 [file Image_5.TIF]
