# Supplementary material for: Identification of Genetic Factors Controlling the Formation of Multiple Flowers Per Node in Pepper (Capsicum spp.)
Source: Front Plant Sci. 2022 May 9;13:884338. doi: 10.3389/fpls.2022.884338 (PMC9125326; doi:10.3389/fpls.2022.884338)
Supplement: Supplementary file 6 [file Data_Sheet_1.docx]

**Supplementary Tables**

**Supplementary Table 1.** Flower number per node of TH RILs

| Line | Node | | | | | |
| --- | --- | --- | --- | --- | --- | --- |
|  | 1st | 2nd | 3rd | 4th | 5th | 6th |
| TF68 | 1.2 | 1.2 | 1.3 | 1.3 | 1.0 | 1.0 |
| Habanero | 5.6 | 3.4 | 3.6 | 5.0 | 2.8 | 3.8 |
| TH-1 | 2.3 | 2.7 | 3.0 | 2.3 | 2.3 | 3.3 |
| TH-2 | 2.0 | 1.7 | 3.0 | 2.0 | 1.7 | 3.0 |
| TH-3 | 1.0 | 1.0 | 2.0 | 1.0 | 1.0 | 1.7 |
| TH-5 | 1.0 | 1.0 | 1.3 | 1.0 | 1.0 | 1.3 |
| TH-9 | 1.0 | 1.0 | 1.0 | 1.0 | 1.0 | 1.0 |
| TH-10 | 2.0 | 2.0 | 3.5 | 2.5 | 2.5 | 3.0 |
| TH-11 | 1.0 | 1.0 | 1.0 | 1.0 | 1.0 | 1.0 |
| TH-13 | 1.0 | 1.3 | 2.0 | 1.0 | 1.3 | 2.3 |
| TH-14 | 1.0 | 1.0 | 1.3 | 1.0 | 1.0 | 1.0 |
| TH-15 | 1.0 | 1.7 | 1.0 | 1.0 | 1.0 | 1.3 |
| TH-16 | 1.0 | 1.0 | 2.0 | 1.0 | 1.7 | 1.7 |
| TH-19 | 1.0 | 2.0 | 2.0 | 1.7 | 2.3 | 2.0 |
| TH-21 | 2.0 | 1.3 | 2.0 | 2.0 | 1.0 | 1.3 |
| TH-22 | 1.7 | 1.0 | 1.5 | 1.7 | 1.3 | 1.0 |
| TH-23 | 1.0 | 1.0 | 1.0 | 1.0 | 1.0 | 1.0 |
| TH-24 | 1.0 | 1.0 | 1.0 | 1.0 | 1.0 | 1.0 |
| TH-25 | 1.0 | 1.0 | 1.3 | 1.0 | 1.0 | 1.7 |
| TH-26 | 1.0 | 1.0 | 1.0 | 1.0 | 1.0 | 1.0 |
| TH-27 | 1.3 | 1.0 | 2.7 | 1.3 | 1.0 | 2.0 |
| TH-28 | 1.3 | 1.0 | 2.0 | 1.7 | 1.0 | 1.7 |
| TH-29 | 1.7 | 2.0 | 2.0 | 1.3 | 2.0 | 1.7 |
| TH-30 | 1.0 | 1.0 | 1.0 | 1.0 | 1.3 | 1.0 |
| TH-32 | 1.3 | 1.0 | 1.0 | 1.3 | 1.0 | 1.0 |
| TH-36 | 2.0 | 2.0 | 2.0 | 2.0 | 1.3 | 2.0 |
| TH-37 | 2.0 | 2.3 | 1.7 | 2.0 | 2.3 | 1.3 |
| TH-39 | 2.0 | 1.3 | 2.0 | 2.0 | 1.7 | 2.0 |
| TH-40 | 1.0 | 1.3 | 1.0 | 1.0 | 1.0 | 1.0 |
| TH-41 | 3.0 | 2.3 | 2.3 | 2.7 | 2.0 | 2.3 |
| TH-43 | 1.3 | 1.0 | 1.7 | 1.3 | 1.0 | 1.3 |
| TH-44 | 2.0 | 1.7 | 2.3 | 2.0 | 2.3 | 2.3 |
| TH-45 | 2.0 | 2.0 | 1.5 | 2.3 | 2.0 | 2.7 |
| TH-46 | 2.3 | 3.0 | 2.3 | 2.7 | 3.0 | 3.0 |
| TH-47 | 2.7 | 2.3 | 2.3 | 3.0 | 2.3 | 2.3 |
| TH-48 | 1.0 | 1.0 | 1.7 | 1.0 | 1.0 | 1.0 |
| TH-49 | 1.0 | 1.0 | 1.3 | 1.0 | 1.3 | 1.3 |
| TH-50 | 1.0 | 1.0 | 1.0 | 1.0 | 1.0 | 1.0 |
| TH-51 | 1.5 | 1.5 | 1.0 | 2.0 | 1.0 | 1.0 |
| TH-52 | 1.3 | 1.0 | 1.0 | 1.7 | 1.0 | 1.3 |
| TH-53 | 1.0 | 1.0 | 1.0 | 1.0 | 1.3 | 1.0 |
| TH-54 | 1.0 | 1.0 | 1.0 | 1.0 | 1.0 | 1.0 |
| TH-56 | 1.3 | 1.7 | 1.0 | 1.7 | 1.3 | 1.3 |
| TH-57 | 1.0 | 1.0 | 1.0 | 1.3 | 1.0 | 1.7 |
| TH-58 | 1.0 | 1.0 | 1.0 | 1.0 | 1.3 | 1.0 |
| TH-59 | 1.7 | 1.7 | 2.7 | 1.7 | 1.7 | 2.3 |
| TH-60 | 1.0 | 1.3 | 1.3 | 1.0 | 1.3 | 1.3 |
| TH-61 | 1.3 | 2.0 | 1.7 | 1.7 | 2.0 | 1.7 |
| TH-62 | 1.0 | 1.3 | 1.3 | 1.0 | 1.3 | 1.7 |
| TH-63 | 1.3 | 1.0 | 1.3 | 1.3 | 1.0 | 1.3 |
| TH-66 | 1.0 | 1.7 | 3.3 | 1.3 | 2.0 | 3.0 |
| TH-67 | 2.0 | 2.3 | 2.3 | 2.0 | 2.3 | 2.7 |
| TH-68 | 1.0 | 1.0 | 1.0 | 1.0 | 1.0 | 1.0 |
| TH-69 | 1.0 | 1.0 | 1.0 | 1.0 | 1.0 | 1.0 |
| TH-70 | 1.0 | 1.0 | 1.3 | 1.0 | 1.3 | 1.7 |
| TH-71 | 2.0 | 1.0 | 2.3 | 2.3 | 1.0 | 2.0 |
| TH-72 | 1.0 | 1.0 | 1.0 | 1.0 | 1.0 | 1.0 |
| TH-73 | 2.3 | 1.7 | 1.7 | 2.3 | 2.0 | 1.5 |
| TH-74 | 1.3 | 1.0 | 1.7 | 1.3 | 1.3 | 1.7 |
| TH-75 | 4.0 | . | . | . | . | . |
| TH-76 | 1.3 | 1.0 | 1.3 | 1.3 | 1.0 | 1.3 |
| TH-77 | 1.0 | 1.0 | 1.0 | 1.0 | 1.0 | 1.0 |
| TH-78 | 1.3 | 1.0 | 1.0 | 1.3 | 1.0 | 1.0 |
| TH-79 | 1.0 | 1.0 | 1.0 | 1.0 | 1.0 | 1.0 |
| TH-81 | 2.0 | 1.0 | 1.3 | 2.0 | 1.0 | 1.3 |
| TH-83 | 1.0 | 1.0 | 1.3 | 1.0 | 1.0 | 1.0 |
| TH-84 | 1.0 | 1.0 | 1.0 | 1.0 | 1.0 | 1.0 |
| TH-85 | 4.3 | 2.7 | 2.3 | 3.7 | 2.7 | 2.7 |
| TH-88 | 1.0 | 1.0 | 1.0 | 1.0 | 1.0 | 1.0 |
| TH-89 | 1.0 | 1.0 | 1.0 | 1.0 | 1.0 | 1.0 |
| TH-90 | 1.0 | 1.0 | 1.0 | 1.0 | 1.0 | 1.3 |
| TH-91 | 2.3 | 2.7 | 2.7 | 2.7 | 1.7 | 3.0 |
| TH-93 | 1.0 | 1.0 | 1.0 | 1.0 | 1.0 | 1.0 |
| TH-94 | 1.0 | 1.0 | 1.3 | 1.0 | 1.0 | 1.3 |
| TH-95 | 1.0 | 1.0 | 1.0 | 1.0 | 1.0 | 1.0 |
| TH-96 | 1.7 | 2.7 | 2.3 | 2.7 | 2.0 | 2.7 |
| TH-97 | 1.3 | 2.7 | 3.0 | 1.3 | 2.0 | 3.0 |
| TH-98 | 2.5 | 1.5 | 1.0 | 2.3 | 1.7 | 1.7 |
| TH-99 | 1.0 | 1.0 | 1.0 | 1.0 | 1.7 | 1.0 |
| TH-100 | 1.0 | 1.0 | 1.0 | 1.0 | 1.3 | 1.0 |
| TH-101 | 1.0 | 1.0 | 1.0 | 1.0 | 1.0 | 1.0 |
| TH-102 | 1.0 | 1.0 | 1.0 | 1.0 | 1.0 | 1.0 |
| TH-113 | 1.0 | 1.0 | 1.0 | 1.0 | 1.0 | 1.0 |
| TH-114 | 1.0 | 1.0 | 1.0 | 1.0 | 1.0 | 1.3 |
| TH-116 | 2.0 | 1.7 | 2.5 | 2.3 | 1.7 | 3.7 |
| TH-117 | 1.0 | 1.0 | 1.0 | 1.0 | 1.0 | 1.0 |
| TH-118 | 1.0 | 1.0 | 1.0 | 1.0 | 1.3 | 1.7 |

**Supplementary Table 2.** Primers used in SCAR marker design and sequence variation analysis for *SP5G.*

| Primer name | Primer sequence (5ʹ to 3ʹ) | Position (Dempsey Chr. 05) |
| --- | --- | --- |
| FT_F | ATGCCAAGAGATCCTTTAATT | 241,815,139–241,815,159 |
| FT_R | TTATAGACGACGACCACCAGT | 241,818,433–241,818,453 |
| FT_F2 | ATCGTAGCTAAACCCCAAACAC | 241,814,926–241,814,947 |
| FT_R3 | CAAGCAAGTTAGGTGCCTTTTC | 241,817,637–241,817,658 |

**Supplementary Table 3.** Haplotype blocks estimated by GBS of the CSHL population.

| Chr. | Number of  SNPs* | Avg. distance  between SNPs (bp) | Number of SNPs  grouped into blocks | Number of  blocks | Average  block size (kbp) | Avg. number of  SNPs per LD block |
| --- | --- | --- | --- | --- | --- | --- |
| 1 | 18,640 | 17,854 | 10,665 | 726 | 365.78 | 14.69 |
| 2 | 9,904 | 17,749 | 5,817 | 381 | 373.85 | 15.27 |
| 3 | 15,780 | 18,453 | 9,137 | 511 | 430.63 | 17.88 |
| 4 | 12,267 | 20,247 | 7,225 | 437 | 442.35 | 16.53 |
| 5 | 11,681 | 21,418 | 6,906 | 377 | 466.22 | 18.32 |
| 6 | 12,891 | 19,354 | 7,760 | 471 | 462.03 | 16.48 |
| 7 | 13,842 | 18,991 | 8,475 | 454 | 466.66 | 18.67 |
| 8 | 9,581 | 18,102 | 5,630 | 217 | 483.45 | 25.94 |
| 9 | 13,084 | 20,758 | 8,062 | 476 | 481.5 | 16.94 |
| 10 | 12,068 | 19,899 | 7,498 | 465 | 474.03 | 16.12 |
| 11 | 13,695 | 19,800 | 8,209 | 440 | 477.24 | 18.66 |
| 12 | 13,156 | 19,584 | 7,639 | 418 | 481.58 | 18.28 |
| Total | 156,589 | 19,351 | 93,023 | 5,373 | 450.44 | 17.81 |

*Number of SNPs specifies only filtered SNPs and used for GWAS analysis

**Supplementary Table 4.** Physical locations of 28 SNPs associated with the multiple-flowers trait detected by GWAS analysis.

| Chr. | Physical position (Mbp) | Chr. | Physical position (Mbp) |
| --- | --- | --- | --- |
| 1 | 137.55–140.9 | 6 | 105.06–105.82 |
| 2 | 125.98–126.37 | 7 | 230.54–231.16 |
|  | 129.54–129.78 |  | 240.15–240.62 |
|  | 134.56–135.34 | 10 | 31.07–31.68 |
|  | 142.23–142.35 |  | 89.42–89.84 |
| 3 | 15.56–16.17 |  | 103.71–104.06 |
|  | 162.53–162.64 |  | 167.78–169.41 |
|  | 251.63–254.11 |  | 183.16–183.78 |
| 5 | 126.92–127.61 |  | 186.95–197.02 |
|  | 198.95–200.41 |  | 216.44–216.93 |
|  | 207.88–207.94 | 11 | 3.79–8.56 |
|  | 212.4–215.34 | 12 | 6.84–7.25 |
|  | 221.15–221.76 |  | 206.77–210.47 |
|  | 227.73–228.26 |  |  |
|  | 231.08–237.02 |  |  |

**Supplementary Figure legends**

**Supplementary Figure 1.** Comparison of flowering phenotypes in the parental lines. *Capsicum annuum* TF68 bears a single flower per node (A), *Capsicum chinense* Habanero produces multiple flowers per node (B) that later form fruits (C).

**Supplementary Figure 2.** SNP density (number of SNPs within 1 Mbp windows) in the TH RIL (A) and CSHL (B) populations.

**Supplementary Figure 3.** Comparison of the genetic map calculated from GBS of the TH RILs with the physical map. Bars on the left of the figure represent the genetic map position (cM) while bars on the right indicate the physical map position (bp). On each chromosome, left bars indicate individual linkage groups, and right bars specify the physical location of each marker.

**Supplementary Figure 4.** Manhattan plot of SNPs associated with the multiple-flowers-per-node trait in the CSHL population. QQ plot in the top-right corner shows the expected distribution of association test statistics (x-axis) across the million SNPs compared to the observed values (y-axis).

**Supplementary Figure 5.** Structural variation of *SP5G* in the parental lines (A) and SCAR marker genotyping of *SP5G* in the CSHL population (B). Dark blue arrows indicate exons and grey boxes denote introns. The red dotted line indicates the deletion within the first intron of *SP5G* in the Habanero parental line. Primers used for gene analysis are depicted as black arrows above the gene model. A larger PCR product of 2,737 bp represents the TF68-type allele, while a smaller PCR product of 2,148 bp characterizes the Haba-type allele.
