# Supplementary material for: Identification of Genetic Factors Controlling the Formation of Multiple Flowers Per Node in Pepper (Capsicum spp.)
Source: Front Plant Sci. 2022 May 9;13:884338. doi: 10.3389/fpls.2022.884338 (PMC9125326; doi:10.3389/fpls.2022.884338)
Supplement: Supplementary file 7 [file Data_Sheet_2.docx]

**Supplementary Data 1.** Comparison of *MEI2-like 5* protein sequences between ‘CM334’ and ‘PI159236’

CM334 MPMINLSKEKEATPWGICPGSDSILVSSDASLFSSSVPVLLHEKLTLNDDKHGHQSIDDA 60

PI159236 MPMINLSKEKEATPWGICPGSDSIHVSSDASLFSSSVPVLLHEKLTLNDNKHGHQSIDDA 60

************************ ************************:**********

CM334 SPSLKKIHPDVEIDELLDDIENHAIGSLLPDDEDELLAGIMDGFDPSRLPNHTDDLEEYD 120

PI159236 SPSLKKIHPDVEIDELLDDIENHAVGSLLPDDEDELLAGIMDGFDPRRLPNHTDDLEEYD 120

************************:********************* *************

CM334 IFGSGGGFELESDGQEHLNLGISRVSLADPVGSNGAAIYGFSNGGGMVTGEHPLGEHPSR 180

PI159236 IFGSGGGFELESDGQEHLNLGISRVSLADPVGSNGAAIYGFSNGGGMVTGEHPLGEHPSR 180

************************************************************

CM334 TLFVRNINSNVEDSELRALFEQYGDIRTLYTACKHRGFVMISYFDIRAARTAMRALQNKP 240

PI159236 TLFVRNINSNVEDSELRALFEQYGDIRTLYTACKHRGFVMISYFDIRAARTAMRALQNKP 240

************************************************************

CM334 LRRRKLDIHFSIPKDNPSDKDVNQGTLVVFNLDPSVSNDDLRQIFGAYGEIKEIRETPHK 300

PI159236 LRRRKLDIHFSIPKDNPSDKDVNQGTLVVFNLDPSVSNDDLRQIFGAYGEIKEIRETPHK 300

************************************************************

CM334 RHHKFIEYYDVRAAEAALRSLNRSDIAGKRIKLEPSRPGGARRNIILQSNQEPEQDDSWT 360

PI159236 RHHKFIEYYDVRAAEAALRSLNRSDIAGKRIKLEPSRPGGARRNIILQSNQEPEQDDSWT 360

************************************************************

CM334 FRHPLGSSIGNSSPGNWPQFGSPVEHGSTQSPGTSPGFRSLSPTIDNNLHGLASILNPRA 420

PI159236 FRHPLGSSIGNSSPGNWPQFGSPVEHGSTQSPGTSPGFRSLSPTIDNNLHGLASILNPRA 420

************************************************************

CM334 SNTNTLRVAPIGEDRTMNGHADFRNGSNHAAPFPQSHSFPDPNISQFGGTMSSFGASNTN 480

PI159236 SNTNTLRVAPIGEDRTMNGHADFRNGSNHAAPFPQSHSFPDPNISQFGGTMSSFGASNTN 480

************************************************************

CM334 GSAVETLSGPQFLWGSPKLHPQQSNSSSWKTQSSTNSFTFSGQGDRFSLSNHQKSFLSSS 540

PI159236 SSAVETLSGPQFLWGSPKLHPQQSNSSSWKTQSSTNSFTFSGQGDRFSLSNHQKSFLSSS 540

.***********************************************************

CM334 QHHHQHLHHVGSAPSGLPFDRHLGFYPDSSILNTGFRGMGIGPRDGSLMVNYGARTTSNA 600

PI159236 QHHHQHLHHVGSAPSGLPFDRHFGFYPDSSILNTGFRGMGIGPRDGSLMVNYGARTTSNA 600

**********************:*************************************

CM334 GVAIPGNMSDNGSLGFGMMSSQRLSPLFLGNGHFSGHAASGFEGLTERSRTRRVDNNSGN 660

PI159236 GVAIPGNMSDNGSLGFGMMSSQRLSPLFLGNGHFSGHAASGFEGLTERSRTRRVDNNSGN 660

************************************************************

CM334 QMDNKKLFQLDLNRIRSGEDTRTTLMIKNIPNKYTSKMLLAAIDEQHKGTFDFLYLPIDF 720

PI159236 QMDNKKLFQLDLNRIRSGEDTRTTLMIKNIPNKYTSKMLLAAIDEQHKGTFDFLYLPIDF 720

************************************************************

CM334 KNKCNVGYAFINMLSPSLIIPFYEAFNGKKWEKFNSEKVAALAYARIQGKTALVAHFQNS 780

PI159236 KNKCNVGYAFINMLSPSLIIPFYEAFNGKKWEKFNSEKVAALAYARIQGKTALVAHFQNS 780

************************************************************

CM334 SLMNEDKRCRPILFHSESSELGDQIVQEHLSSGCVHIQVSQSNESDLVGSQGSPPEEDPV 840

PI159236 SLMNEDKRCRPILFHSESSELGDQIVQEHLSSGCVHIQVSQSNESDLVGSQGSPPEEDPV 840

************************************************************

CM334 DKLEKT 846

PI159236 DKLEKT 846

******

**Supplementary Data 2.** Comparison of *WOX9* protein sequences between ‘Dempsey’ and ‘PI159236’

Dempsey MASSNRHWPSMFKSKPCNSHHHQWQHDINSSLIQPRPPCNQEERSPEPKPRWNPRPEQIR 60

PI159236 MASSNRHWPSMFKSKPCNSHHHQWQHDINSSLIQPRPPCNPEERSPEPKPRWNPRPEQIR 60

**************************************** *******************

Dempsey ILETIFNSGMVNPPRDEIRKIRARLQEYGQVGDANVFYWFQNRKSRSKHKQRHVMNLLNS 120

PI159236 ILETIFNSGMVNPPRDEIRKIRARLQEYGQVGDANVFYWFQNRKSRSKHKQRHVMNLLNS 120

************************************************************

Dempsey PTASVNQQNYNNDQFFTTTEQPFFFTVQQPVQTHDNSAMTQGFCFPDSTSSSSGLALSEL 180

PI159236 PTASVNQQNYNNDQFFTTTEQPFFFTVQQPVQTHDNSAMTQGFCFPDSTSSSSGLALSEL 180

************************************************************

Dempsey MGISQTHSSSKKAENEKMNLQSQLMSYTVTSTPTTVSPLISTTTIPTISHIQGASLDPNE 240

PI159236 MGISQTPSSSKKAENEKMNLQSQLMSYTVTSTPTTVSPLISTTTIPTISHIQGASLDPNE 240

****** *****************************************************

Dempsey AVGPTRSTVFINDFAFEVGLGPFNVREVFGEDAVLFHSSGEPLITNEWGLTLQPLQHGAF 300

PI159236 AVGPTRSTVFINDVAFEVGLGPFNVREVFGEDAVLFHSSGEPLITNEWGLTLQPLQHGAF 300

*************.**********************************************

Dempsey YYLVRTSTPSTHDI 314

PI159236 YYLVRTSTPSTHDI 314

**************

**Supplementary Data 3.** Comparison of *SP5G* CDS between ‘TF68’ and ‘Habanero’

TF68 ATGCCAAGAGATCCTTTAATTGTTTCTGGAGTTGTTGGAGATGTTGTGGATCCATTCACT 60

Haba ATGCCAAGAGATCCTTTAATTGTTTCTGGAGTTGTTGGAGATGTTGTGGATCCATTCACT 60

************************************************************

TF68 AGGTGTGTGGATTTTGGTGTTGTTTACAACAATAGGGTTGTGGTCTACAATGGATGTGCC 120

Haba AGGTGTGTGGATTTTGGTGTTGTTTACAACAATAGGGTTGTGGTCTACAATGGATGTGCC 120

************************************************************

TF68 TTGAGGCCTTCACAAGTTGTCAATCAACCTAGGGTTGAAATTGGTGGCGACGATCTTCGC 180

Haba TTGAGGCCTTCACAAGTTGTCAATCAACCTAGGGTTGAAATTGGTGGTGACGATCTTCGC 180

*********************************************** ************

TF68 ACTTTTTACACGCTGGTTATGGTAGACCCTGATGCTCCAAACCCTAGCAACCCAAACCTA 240

Haba ACTTTTTACACGCTGGTTATGGTAGACCCTGATGCTCCAAACCCTAGCAACCCAAACCTA 240

************************************************************

TF68 AGGGAGTATCTACACTGGTTGGTCACAGATATCCCCGCAACTACAGGAGCAAACTTTGGC 300

Haba AGGGAGTATCTACACTGGTTGGTCACAGATATCCCCGCAACTACAGGAGCAAACTTTGGC 300

************************************************************

TF68 AATGAAGTTGTAAGCTACGAGAGCCCACGTCCATCAATGGGAATCCATCGCTATATCTTC 360

Haba AATGAAGTTGTAAGCTACGAGAGCCCACGTCCATCAATGGGAATCCATCGCTATATCTTC 360

************************************************************

TF68 GTGTTGTATCGACAATTGGGCCGCGAGGCGATCAATGCGCCAGACATAATTGATTCACGC 420

Haba GTGTTGTATCGACAATTGGGCCGCGAGGCGATCAATGCGCCAGACATAATCGATTCACGC 420

************************************************** *********

TF68 CAGAATTTTAACACCAGAGATTTTGCTAGGTTTCATAATCTTGGTGTTCCTGTTGCTGCT 480

Haba CAGAATTTTAACACCAGAGATTTTGCTAGGTTTCATAATCTTGGTGTTCCTGTTGCTGCT 480

************************************************************

TF68 GTTTACTTCAATTGCAATAGGGAAGGTGGTACTGGTGGTCGTCGTCTATAA 531

Haba GTTTACTTCAATTGCAATAGGGAAGGTGGTACTGGTGGTCGTCGTCTATAA 531

***************************************************
